# Recent Progress on Metal-Enhanced Photocatalysis: A Review on the Mechanism

**DOI:** 10.34133/2021/9794329

**Published:** 2021-06-10

**Authors:** Ming Fang, Xiaoli Tan, Zhixin Liu, Baowei Hu, Xiangke Wang

**Affiliations:** ^1^College of Environmental Science and Engineering, North China Electric Power University, Beijing 102206, China; ^2^School of Life Science, Shaoxing University, Shaoxing 312000, China

## Abstract

Metal-enhanced photocatalysis has recently received increasing interest, mainly due to the ability of metal to directly or indirectly degrade pollutants. In this review, we briefly review the recent breakthroughs in metal-enhanced photocatalysis. We discussed the recent progress of surface plasmon resonance (SPR) effect and small size effect of metal nanoparticles on photocatalysis; in particular, we focus on elucidating the mechanism of energy transfer and hot electron injection/transfer effect of metal nanoparticles and clusters while as photocatalysts or as cophotocatalysts. Finally, we discuss the potential applications of metal-enhanced photocatalysis, and we also offer some perspectives for further investigations.

## 1. Introduction

Along with the accelerated development of human society, increasing energy concerns and environmental deterioration have become the most serious problems facing human beings [1–3]. To mitigate these problems, great efforts have been made. Among the various methods, photocatalysis is an effective way that can not only produce clean energy (such as H_2_) but also fix environmental problems, and most importantly, all these transformations are driven by inexhaustible solar energy [4]. Photocatalysis is a green energy technology without consuming fossil fuels. The power of the solar light is approximately 100 mW/cm^2^ (10^7^ photons per square centimeter), meaning that there are approximately 100 photons to reach one atom in one second. Many of the used semiconductor photocatalysts could only absorb photons higher than the bandgap. However, the bandgaps of the usually used TiO_2_ and ZnO are approximately 3.6 and 3.2 eV, respectively, which result in the absorption of these materials being usually in the ultraviolet region (the ultraviolet light only accounts for 5% of the whole solar spectrum). Therefore, it is of great interest to find ways to improve the absorption ability, both in broadening the wavelength range and in improving the absorption intensity. Many strategies have been developed to construct highly efficient photocatalysts, such as bandgap engineering, doping, morphology and size modulation, and loading with metal or nonmetal particles [5, 6]. Among the various methods, the combination of semiconductors with metal nanoparticles, especially with noble metal nanoparticles, has recently aroused great attention.

**Figure 1:**
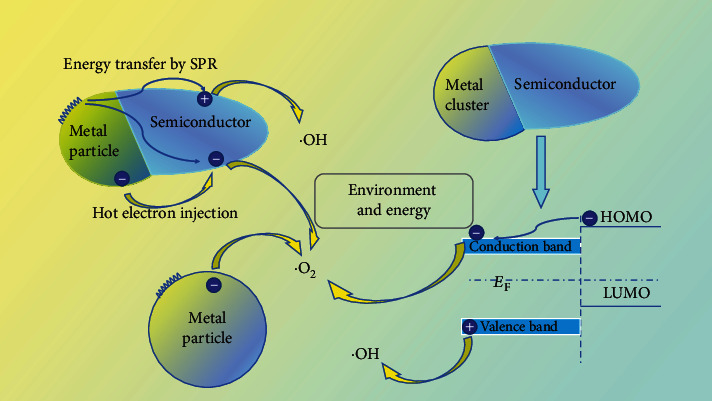
The schematic diagram on the mechanism of metal-enhanced photocatalysis.

The light energy harvesting ability is related to the absorption ability, which is dependent on the atomic absorption cross-section. In general, the atomic absorption cross-section is approximately 10^−17^~10^−16^ cm^2^ [7], while for the noble metal atoms, it is approximately 10^−13^~10^−12^ cm^2^ [8]. This means the noble metal atoms can absorb light approximately 10^4^~10^5^ times more than the common ones can, obtaining more energy and inducing electric fields with an intensity of 2~6 orders over that of the incident light. The absorbed light energy then can be used to enhance photocatalysis and has been investigated by many researchers. According to the different enhancement mechanisms of metals on the semiconductor photocatalysts, three metal forms could usually be considered: doping ions, nanoclusters, and plasmonic nanoparticles. The doping effect has been reviewed well, and also due to the space limitation, in this review, we mainly focus on reviewing the recent progress on the metal-enhanced photocatalysis by zero-valent metal particles, especially on the transfer mechanism of electrons or holes, between the materials and the target molecules. If the readers are interested in the dopant, they can search for some recent references [9–11]. Although the plasmonic nanoparticles have the same zero valence state as the metal clusters, the physicochemical mechanisms are very different due to the size (see Figure 1). For metal nanoparticles with a size larger than the de Broglie wavelength (or Fermi length; for convenience, in this review, we take it as 2 nm), especially those of the noble metals, they can be considered large, heavy, and positive nuclei wrapped by a free electron gas. When the metal nanoparticles are placed in the electromagnetic field of light, the free electrons may absorb and store the light energy as energetic electrons. Then, the electrons intend to deviate from the equilibrium position, while the repulsion among the electrons causes the electrons to keep away from each other. The spatial density of the electrons is rearranged with the electromagnetic field. Finally, the electrons oscillate together under the interactions at a certain resonant frequency, which is named surface plasmon resonance (SPR) [12]. This energy could be transferred to the neighboring material, which can further greatly improve the Raman signals, fluorescence, photocatalysis, single-molecule spectroscopy properties, etc., and thus foster many new powerful analytical methods and applications. However, for metal particles with a size smaller than 2 nm, which are usually called nanoclusters, the energy bands begin to split and form some new energy levels due to the quantum size effect, and the behavior of a metal nanoparticle is more like that of a molecular cluster without exhibiting the SPR effect anymore. The metal nanoclusters could transfer the ions with high energy to the recent hybridizer like a semiconductor, which could also greatly enhance photocatalysis.

## 2. Plasmonic Photocatalysis

The SPR effect has been observed in many metal particles; however, the most investigated ones are the noble metals, such as Ag, Au, and Cu, which have high chemical stability and photostability. To induce the SPR, the carrier density should be over 10^19^ cm^−3^ [13]. The corresponding SPR of noble metals is usually located in the visible light range and has a free electron density of 10^22^–10^23^ cm^−3^ [13]. For the common metals, such as Al, Pb, In, Sn, Cd, and Hg, the SPR peaks are usually located in the ultraviolet light region and are untunable compared to that of the noble metals. The common metals are usually more active than the noble metals, making the small particles readily react with oxygen, water, etc. Thus, it is difficult to obtain pure nanoparticles of these metals for measurement.

The SPR-enhanced photocatalysis, also known as plasmonic photocatalysis, was firstly named by Watanabe and coworkers, who embedded Ag nanoparticles into layers of TiO_2_. Under UV illumination, they observed the significant improvement of photocatalytic degradation activity and ascribed this to the enhancement effect of localized SPR via near-field electromagnetic waves [14]. Due to the light concentration effect, the expanding light response range, and the effective energy transfer ability to the neighbors, SPR-enhanced photocatalysis has been an investigation of focus recently [15].

### 2.1. Direct Plasmonic Photocatalysis

Based on the experimental and theoretical computational results, the SPR was proposed to be able to donate energetic electrons into the energy orbitals (states) of available adsorbates, forming negative ion species. If the plasmonic nanoparticles are combined with a semiconductor, the energy may be transferred to the semiconductor, while if it is directly contacted with the degradation object, photocatalysis could also be performed. Therefore, photocatalysis of plasmonic nanoparticles can be achieved in two ways, direct and indirect photocatalysis [16]. Direct photocatalysis is usually achieved only by metal nanoparticles, where the SPs could directly react with adjacent adsorbates. Indirect photocatalysis is that the SPs transfer the light energy to the neighboring semiconductor, polymeric catalyst, etc., which will be discussed later.

In the direct photocatalytic way, the hot electrons are transferred to the surface of metal nanoparticles and can directly react with the surface adsorbate to form negative ions (i.e., transient negative ions), which could rapidly react on the metal surface or move into the solution [17]. The hot electrons produced by the SPR effects on the surface have been observed to be able to induce the dissociation of the adsorbed molecules or form active radicals [17], and CO_2_ has been found to be reduced by gold nanoparticles via a multiple-electron process under plasmonic excitations [18]. A Pt-edged Au nanoprism without a semiconductor has been reported to be able to catalyze hydrogen production under visible light irradiation by Lou and coworkers, and the rate of hydrogen production is 0.167 *μ*mol/h [19]. In these experiments, the electrons transferred from Au nanoparticles to the adsorbates by a one-electron (1e^−^) reduction process, and the photoinduced electron-hole pairs could be generated from the transition of d → sp energy levels or the process of Landau damping. Multiple-electron reactions can also be driven under some appropriate conditions (high photon flux, quick scavenging of holes, or interband excitation) with continuous-wave illumination [16].

Therefore, the direct plasmonic photocatalysis is very different from that of semiconductors [20]:

1. The photocatalysis of metal nanoparticles exhibits a superlinear power law versus light intensity; i.e., the photocatalytic rate is proportional to the intensity of the *n*th power (*n* > 1), while it has a relatively low quantum efficiency for semiconductors at high light intensity [21]
2. Increasing the working temperature increases the quantum efficiency and rate of photocatalysis, which is also low for semiconductors at high temperatures. The photocatalytic rate is exponentially dependent on the working temperature [22, 23]

**Figure 2:**
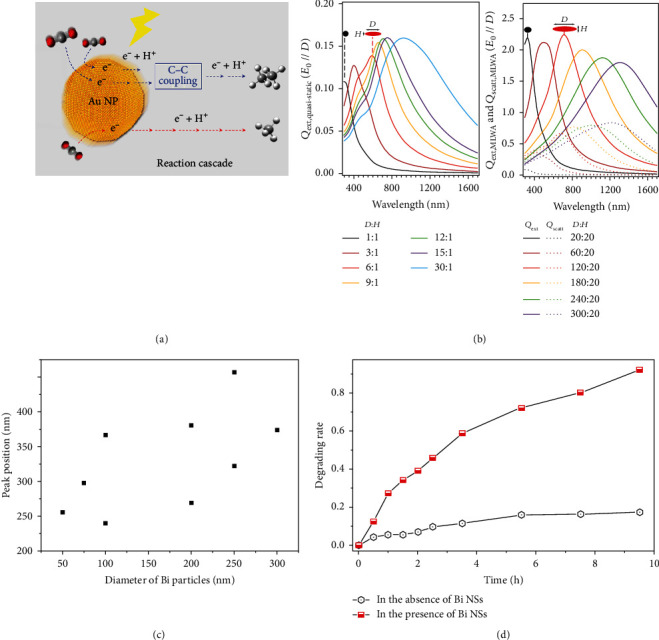
(a) Visible light-driven CO_2_ reduction to hydrocarbons using a plasmonic Au NP photocatalyst [18]. (b) The calculated transverse extinction spectra of Bi nanoparticles in an Al_2_O_3_ matrix: by quasistatic dipolar approximation (left) and by modified long-wavelength approximation (right) [27]. (c) The calculated SPR peaks of Bi nanoparticles in different diameters. (d) The photocatalytic ability of Bi nanospheres to RhB [29].

The direct photocatalysis of the noble metal nanoparticles acts as both the light absorbent and the reaction active spot [24]. The CO_2_ molecules can be photoreducted to hydrocarbons by Au nanoparticles under visible light illumination (Figure 2(a)) [18]. There are approximately 10^11^~10^12^ SPs induced by light per second [25]. The hot electrons may transfer to the 2*π*^∗^ orbital, forming the transient anion. When the excitation rate of SP is lower than the vibration decay rate of O_2_, the photocatalytic rate linearly depends on the intensity of light. Otherwise, there may be an induced ultralinear process. At this time, the relaxation of transient anions may reemit phonons and again excite an SP. This process could repeat again and again, and one phonon may excite 10 SPs. Compared with semiconductor photocatalysis, direct SP-induced photocatalysis has more intriguing aspects [26].

As mentioned above, Bi nanoparticles could also induce the SPR effect. Toudert and coworkers calculated the SPR peaks of Bi nanoparticles. With increasing aspect ratios, the SPR peaks of Bi can be tuned in the range from the ultraviolet light region to the near-infrared light region by modulating the diameter and the height (Figure 2(b)) [27]. The SPR peak positions versus diameters of Bi nanoparticles are shown in Figure 2(c), in which the data were measured from Reference [28]. With increasing particle diameters, the peak positions shift towards the red, and the Bi nanoparticles exhibited photocatalytic activities under 420 and 360 nm irradiation. These works indicate the great application potential of Bi nanoparticles as tunable plasmonic nanostructures, which can also harvest light and transfer energy to the adjacent adsorbates or be used as a photocatalyst directly. However, there are only a few reports related to the utilization of Bi nanoparticles to boost photocatalytic activity by the SPR effect. In a recent experiment, under 280 nm irradiation, the Bi nanoparticles showed a high photocatalytic efficiency towards NO. In another experiment, Wang and coworkers fabricated Bi nanoparticles for the photocatalysis of RhB (Figure 2(d)) [29]. The degradation of RhB was found to be greatly enhanced with the addition of Bi nanoparticles. Additionally, after measuring the XRD curves of the sample after photocatalysis, they confirmed the degradation indeed originated from photocatalysis by Bi nanoparticles wrapped with an oleylamine protection layer.

### 2.2. Indirect Plasmonic Photocatalysis

The hybridized metal nanoparticles have proven to be able to transfer the concentrated energy to the adjacent semiconductors or adsorbed molecules by SPR effect, thus increasing the absorption ability and extending the absorption spectrum (to the visible and near-infrared regions) of the composite. The energy transfer from SPR includes radiative transfer and nonradiative transfer. The nonradiative transfer is considered to lead to the heating effect by SPR that could also contribute to the enhancement of photocatalysis [30]. However, sufficient proof is still lacking, and this topic will not be reviewed here.

#### 2.2.1. The Photocarriers' Lifetime Prolonged by Schottky Barrier Effect

Among the various effective methods to prolong the lifetime of photoinduced electrons and holes, hybridization with metal nanoparticles has aroused great attention. The formed Schottky barrier can effectively prevent the recombination of photoinduced electron-hole pairs to a certain extent. As for the p-type semiconductor, the situation is different. The carriers in the semiconductor are the holes. However, no matter what the carrier is, the Schottky barrier could prolong the lifetime of photocarriers [31], which is favorable for improving photocatalysis.

**Figure 3:**
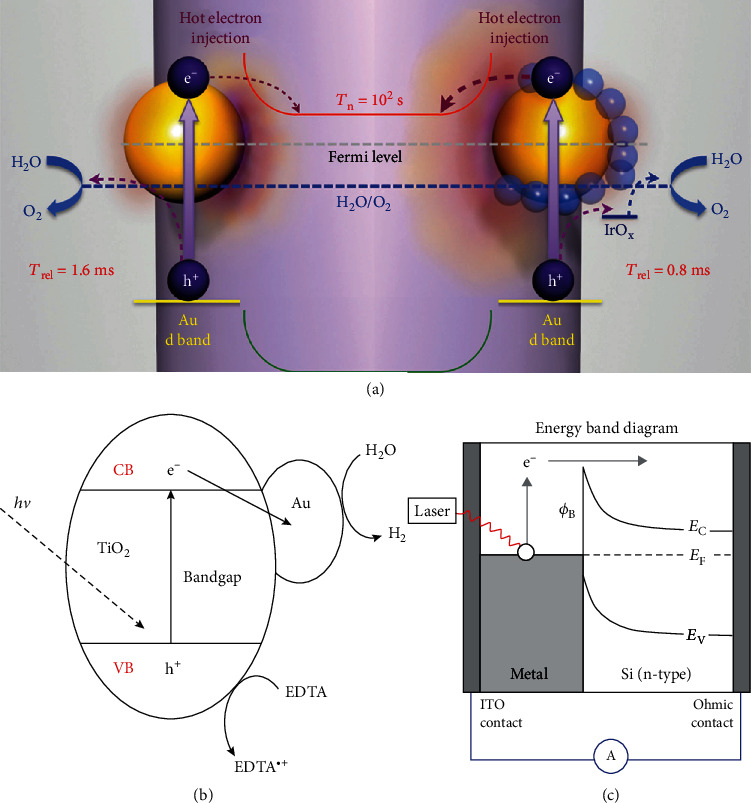
(a) The variation in the interface band structure of Cu_2_O/Ag without and with light irradiation [32]. (b) The photocatalytic activity of Au/TiO_2_ [35]. (c) The interface of an optical antenna-diode [37].

In a ternary photocatalyst of TiO_2_/Au/IrOx (Figure 3(a)) [32], the electrons in the d band of Au dominate the plasmonic hot electrons and can cross the Schottky barrier by a tunneling process, instead of a ballistic traveling process, to TiO_2_. Then, the hot electrons transfer into the conduction band of TiO_2_. On the other hand, as the surface of Au was further combined with IrOx nanoparticles, the relaxation time of holes may be reduced from 1.6 to 0.8 ms, due to the conduction band bottom of IrOx being positioned between the oxidation level of water and the d band of Au. From this process, the lifetime of hot electrons is prolonged, and the transport speed of holes is accelerated, which could thus greatly enhance the photocatalytic activities.

Wei et al. fabricated rGO wrapped octahedral Ag-Cu_2_O heterostructures and investigated the interface band structure of Cu_2_O/Ag without or with light irradiation [15]. After combining with Ag nanoparticles, a metal-semiconductor heterostructure was first formed at the interface. The work function of Ag is 4.10 eV, which is lower than that of Cu_2_O (4.41 eV), making electrons transfer from Ag to Cu_2_O. This process continued until both Fermi levels reached the same level at approximately 4.30 eV. Under light irradiation, the photoelectrons transferred from the conduction band of Cu_2_O to the Ag nanoparticles, thus decreasing the recombination rate of electron-hole pairs and enhancing the photocatalytic activity greatly.

#### 2.2.2. The Injection of Photocarriers

In some recent research, the SPR-induced hot electrons are found to be able to inject into the conduction band of an adjacent semiconductor for diverse reactions, such as reactive oxygen species generation, water splitting, and pollutant degradation [33, 34]. Although the Schottky barrier can prevent the recombination of electron-hole pairs, if the electrons have sufficient energy to overcome the barrier, they can transfer from the metal nanoparticle to the semiconductor. After combining with the metal particles, due to the coupling of light with SPs, the light energy could be concentrated on the metal surface to produce the energetic (hot) electrons, which can participate in the photocatalytic reaction on the metal surface with a lifetime of approximately several tens of fs. However, for the energetic electrons with sufficient energy, they can overcome the Schottky barrier and inject into the conduction band of the semiconductor (Figure 3(b)) [35]. Approximately 1 to 4 eV of energy is evaluated to be able to transfer to one electron from the SPs of Au and Ag [36]. The energy transfer of SPs to electrons is mainly by the Landau damping process via jumping to the transient populated electronic energy levels above the Fermi level to be hot electrons (Figure 3(c)) [37]. The process of electron injection mediated by SPs is achieved in <240 fs [38]. After combining with noble metal, the lifetime of hot electrons increased by 1-2 orders of magnitude compared with that in pure TiO_2_ because of the formation of a Schottky barrier [31]. The long-lived electrons could greatly improve the photocatalytic activity of the semiconductors.

To prove the “hot” electron injection effect, Xu and coworkers utilized an ultrathin SiO_2_ shell coating on Au to test the reaction of “hot” electrons with p-nitrothiophenol (pNTP) (Figure 3(b)) [39]. The pNTP can dimerize into a dimer of DMAB when the hot electrons transfer to the molecule. However, after shielding by the SiO_2_ nanolayer, the corresponding *β*(C-H) Raman vibration peak of DMAB disappeared. Therefore, it could be concluded that the “hot electron” transfer effect can happen as the noble metal particles are directly contacting with a semiconductor, even if the energy of the metal SPR is lower than the bandgap of the semiconductor.

For water splitting, the conduction bands of the semiconductors are usually located in the range from -1.0 to 0 V and the valence bands in the range from 2.0 to 3.5 V, in which the bandgap is a little smaller or comparable to the energy of hot electrons. This means the hot electrons can transfer to the semiconductors' conduction band. Therefore, hot electrons can execute the photocatalytic reactions on the semiconductor side. Lou et al. modified graphene oxide by Au and Pt, which showed a high hydrogen production rate (1000 *μ*mol g^−1^·h^−1^). By systematically investigating the absorption spectra of different samples, they confirm the electron transfer in photocatalytic hydrogen production is due to the SPR effect of Au triangular nanoprisms [40].

Compared with noble metals, Bi is inexpensive and has the SPR effect. Therefore, Bi nanometal particles could also be used as a cophotocatalyst [41]. In the work by Dong and coworkers [41–43], they hybridized Bi metal nanoparticles on the flower-like Bi/(BiO)_2_CO_3_. By modulating the ratio of the metal to the semiconductor, they obtained a photocatalyst that could remove 58% NO; while under the same conditions, the removal rate of pure (BiO)_2_CO_3_ is 22% and is also higher than those of N/C-doped TiO_2_, C_3_N_4_, BiOBr, and Ag/(BiO)_2_CO_3_. The enhancement has been ascribed to the synergistic effect of the SPR of Bi metal nanoparticles and the transfer of electrons from Bi to (BiO)_2_CO_3_. They also added the Bi nanoparticles to g-C_3_N_4_, BiOIO_3_, and TiO_2_ and found the Bi nanoparticles could greatly enhance the absorption in the visible light region, making the composite a visible light response photocatalyst [43, 44].

Most of the reported plasmonic photocatalysts were done in an n-type semiconductor. However, to comprehensively understand the carriers' relaxation dynamics and the utilization of light energy, the investigation on the hot hole injection to the valence band of a p-type semiconductor is needed. Therefore, Tagliabue et al. hybridized Au nanoparticles (7.3 ± 2.4 nm) onto a p-GaN substrate [45]. The size of the Schottky barrier is 1.1 eV to ensure the successful injection of near 90% hot holes to the valence band of GaN. In another work, the hot holes created by Ag particles were proposed to attack citrate into an unstable acetone-1,3-dicarboxylate and finally into acetone and acetyl acidic acid [46].

#### 2.2.3. The Resonance Energy Transfer by SPR

In addition to the hot electron injection, which occurs while the metal nanoparticles are usually in close contact with the semiconductor, the energy can also be transferred to the semiconductor by energy resonance without contact. The resonance energy transfer can be achieved by the electromagnetic effect [42]. This means the energy of the hot electrons may propagate to the semiconductor by a noncontact resonance energy transfer (electromagnetic resonance) process.

To illuminate the resonance energy transfer, a spacer (which should be thin and nonconductive, such as SiO_2_ or Al_2_O_3_) to prevent hot electron injection between the plasmonic metal nanoparticles and the semiconductors is usually used while investigating the energy transfer to enhance the fluorescence and Raman signals. Govorov and coworkers found that after coating a thin SiO_2_ on Au nanoparticles, the electric field at the surface over that of the incident light was calculated to be 85 times that of a 4 nm shell and 142 times that of a 2 nm shell surface, and the Raman scattering intensity was enhanced by 10^7^~10^8^ times [47]. This means the electromagnetic resonance can be a way to transfer energy from the plasmonic metal nanoparticles to the semiconductor [7]. This process is definitely different from the hot electron injection process, although they both are generated by the SPR.

**Figure 4:**
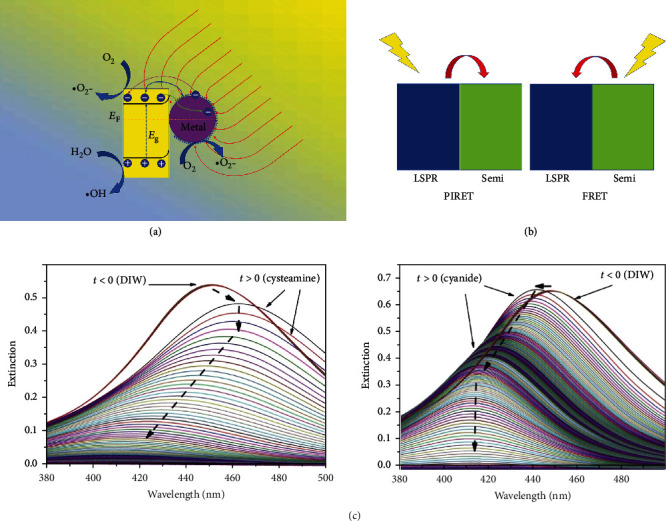
(a) The photocatalytic enhancement mechanism of the SPR on the semiconductor. (b) The illustration of the energy transfer in PIRET and FRET. (c) Excitation spectra of the Ag NP film dissolved by 0.1 mM cysteamine and cyanide, respectively [67].

From the above discussion, for the metals that can generate SPR under light excitation, the electrons and holes may have very high kinetic energies (which are named hot holes and hot electrons, respectively). The photocatalytic enhancement mechanism of SPR on the semiconductor can be concluded in Figure 4(a). After contact with plasmonic metal nanoparticles, the hot electrons in the conduction band of the semiconductor may transfer to the surface of the metal, forming the Schottky barrier (I). The plasmonic metal nanoparticles have the ability to harvest light energy with a large cross-section and can transform into hot electrons, which can further transfer the energy to the adjacent semiconductor in two ways, resonance energy transfer (II) and hot electron injection (III). The former usually dominated in the indirect contact process, and the latter dominated in the direct contact process [48]. The hot electrons on the surface of plasmonic metal nanoparticles and semiconductors could also directly react with soluble oxygen in the solution to form reactive radical ^·^O_2_^−^.

The dipole of the localized SPR can transfer the resonance energy to the electron-hole pairs of the semiconductor. The distance between the plasmonic metal nanoparticles and the semiconductors has an important influence on the energy transfer of the SPR, and the proper distance between the metal nanoparticles and the semiconductors is approximately 1 to 10 nm. At smaller distances, the energy loss is very high due to the nonradiative Förster resonance energy transfer (FRET) process [49].

The FRET is the dipole-dipole coupling effect in an incoherent system with downward energy transfer. Liu et al. [50] raised a modified mechanism of plasmon-induced resonance energy transfer (PIRET, Figure 4(b)) from FRET based on some previous works [50–52]. A four-level system composed of two acceptor levels and two donor levels is proposed. The PIRET is a coherent system, and the population can transfer to the semiconductor after excitation by the plasmon. However, for the FRET, the population transfers to the plasmon after exciting the semiconductor. The dephasing time determines the efficiency of the population transfer. In the dipole-dipole coupling system, the population transfers to one. This means if the dephasing time of the plasmon is short, the FRET is efficient; otherwise, the PIRET is efficient. Additionally, the measured energy transfer efficiency by PIRET depends on the distance as 1/*R*^4^, which is clearly different from that in the FRET of 1/*R*^6^. They fabricated an Au@SiO_2_@Cu_2_O composite material and found the PIRET can transfer the most energy in 10 ps.

To increase the ability of metals on the enhancement of photocatalysis, we here list some features of the plasmonic nanoparticles that may be considered to explain the mechanism:

1. Plasmonic nanostructures can induce a very strong electric field under photoirradiation, which may be several orders of magnitude over that of the incident light [53]. The intensity of the electric field induced by the plasmon (*E*_SP_) varied with the distance from the surface of the nanoparticles, increased exponentially until the highest intensity at approximately 20-30 nm, and further decreased linearly
2. For SPR, the metal nanoparticle size is usually smaller than or comparable to the wavelength of the incident light
3. The plasmonic nanoparticles can be considered a light concentrator, which can focus the incident photons with a very large absorption cross-section than the common atoms to boost the generation and suppress the recombination of the electron-hole pairs
4. The capability of the plasmonic metal nanoparticles to absorb the light can be easily enhanced by modulating their morphology, particle size, etc., over a large range
5. The SPR could decay into “hot” electrons and then transfer to the adjacent semiconductors/molecules to participate in the photocatalytic reactions and thus improve the photocatalytic activity of the composite [54]
6. Low-energy photons can also be absorbed by plasmonic nanoparticles by a two-photon absorption process [55]
7. Photoelectrons on the surface of noble metal nanoparticles have a higher density than that on the semiconductor [56]
8. The most efficient irradiation wavelength was usually at the SPR peak [57]

The biggest difference between the energy transfer of the SPR and the photoinduced electron transfer lies in the different laws relying on the distance. It is well known that the efficiency of energy transfer of SPR goes as the sixth power of the dipole separation, and the effective distance is approximately 1~20 nm [42]. However, for the electron transfer, the dependence on the distance is exponential and the phenomenon tends to be effective at shorter distances, on the order of 10 angstroms, due to the fluorescence of the acceptor being quenched or not observed because the acceptor is converted into a radical anion [58].

#### 2.2.4. The Structural Factors of Materials to Influence the Plasmonic Photocatalysis

In addition to the abovementioned aspects, there are still some ways related to photocatalytic activity. The propagation of SPR may have preferential direction for the different crystal facets with different surface atomic arrangements for semiconductors, while combination may dominantly influence the light absorption ability and the energy transfer capability; thus, the photocatalytic activity could be modulated by exposing different crystal facets [59]. The contact interface may provide for the tunneling of the hot carriers transferring in a metal-semiconductor system [60, 61]. They have an important influence on photocatalysis [62]. The atomic arrangement and contacting way at the interface may greatly influence the charge separation, energy transfer between metal nanoparticles and semiconductors, and absorption ability of the composite and subsequently influence the photocatalytic selectivity and activity [59].

In a recent investigation, Li and coworkers [63] reported the influence of crystal facets on the propagation of hot electrons. They modified Bi nanoparticles on the (010) and (001) facets of BiOBr nanosheets. It was found that the Bi element promotes the hot carrier transfer by the SPR effect. At the (010) facet of BiOBr, electrons were found to prefer to transfer along [Bi_2_O_2_]^2+^ → Bi metal → Br^−^, which means the (010) facet favors photocatalysis by enhancing the electron transfer and charge separation. These results could also be verified in alternative noble metal (Au and Ag) and semiconductor (BiOI and BiOCl) composite systems. This provides an alternative way to enhance photocatalysis by promoting the charge transfer at the interface between a metal and a semiconductor.

The morphology, size, and component of metal nanoparticles can be modulated to improve the ability of SPR on enhancement [36, 64, 65]. It is reported that SPs of particles with a large diameter (Ag, >50 nm) may be subject to the radiative scattering of resonant photons, and the small-diameter particles (<30 nm) are subject to the energetic charge carriers [66]. From a recent report on photocatalytic hydrogen production, the best diameter of Au nanoparticles to hybridize with CdS is 16 nm, which can enhance the photocatalytic efficiency by 405 times after combining [39]. The authors ascribed this to the highest total electromagnetic field enhancement of 16 nm Au because a smaller size may weaken the SPR intensity and a larger one may decrease the quantity of noble metal particles under the same load. Although the explanation still lacks efficient evidence, the experiments still tell us the enhancement is not monotonically reliant on the size of the particles. To demonstrate this, more powerful proofs are needed.

The ability of metal nanoparticles to absorb light (especially the peak position) could also be affected by the matter they are combined with or that which is adsorbed on the surface. The combination with an electron donor may lead to the blueshift of the SPR peak for noble metal particles, while that with an electron acceptor may result in a redshift (see Figure 4(c)) [67]. Two close metal particles can severely enhance the electronic field. The intensity of the electric field around the surface of a single particle is approximately 10^3^ times that of the incident light. However, for two nanoparticles separated by 1 nm, the intensity of the electric field increases to over 10^6^ times that of the incident light.

The structure of the composite should also be considered while designing a highly efficient photocatalyst. Due to the coverage of noble metals, some researchers have confirmed the Ag particles are easily leached out and decrease the number of surface active sites, and the chemical corrosion and photocorrosion can inhibit the activity of the photocatalyst [68, 69]. Therefore, the core-shell-structured hybrid photocatalyst attracted attention. By embedding the noble metal nanoparticles in the semiconductors, the noble metals could be protected from aggregation and chemical corrosion and can maximize the intimate surface with semiconductors, which may thus maximize the effects of the SPR, Schottky barrier, coupling of the energy band, etc. Inspired by the above ideas, Kadam and coworkers [68] fabricated an Ag-core/ZnO-shell nanostructure. By fitting with the Langmuir-Hinshelwood model, the rate constant was found to be four times higher than TiO_2_ (P25) and six times higher than ZnO. They proposed the effective Schottky effect and enhanced electron transfer from ZnO to Ag may be responsible. However, the mechanism explaining the photocatalysis of this structure still has some issues, such as the embedded Ag in ZnO may lose the chance to contact the pollutant, which means the electrons in Ag could not directly interact with the pollutant.

Although the binary composites of metal nanoparticles with semiconductors have shown great enhancement in photocatalysis, pursuing a more efficient photocatalyst is still full of challenges. How to effectively utilize the separation effect at the interface and improve the light utilization can both be achieved by modulation of the composition, morphology, size, etc. Recently, the hybridization of a ternary composite with different functional parts has indicated great application potential. In recent research, Xu and coworkers hybridized Bi nanoparticles with BiOCl and P25 forming a ternary photocatalyst. The Bi nanoparticles broadened the absorption spectrum of BiOCl and P25 from the ultraviolet region to the visible light region due to the LSPR effect. The composite shows a very high photocatalytic efficiency of 53.6 times compared with that of Bi/BiOCl and 23.9 times of P25, which has been ascribed to the LSPR effect of Bi and the heterojunction between BiOCl and P25 [70].

Apart from the traditional nanosemiconductors, some newly investigated materials, such as copper-based metal-organic frameworks (MOF), have also been introduced as a component of a hybrid photocatalyst. In a recent research, Sofi and coworkers reported the construction of a visible light-driven photocatalyst by combining Ag nanoparticles with Ag_3_PO_4_ and a copper-based MOF [71]. The authors proposed a Z-scheme figure to explain the high performance in photocatalysis. The photoelectrons transferred from Ag_3_PO_4_ to Ag and finally to the MOF and formed ^·^O_2_^−^ by reacting with the solvated oxygen. In the other route, the left H^+^ in the valence band of Ag_3_PO_4_ could also react with H_2_O to form ^·^OH, which can together with ^·^O_2_^−^ degrade organic pollutants. By such a mechanism, the separation of electron-hole pairs is more effective, and their lifetime is also prolonged.

In another ternary complex photocatalyst, an Ag@AgCl/ZnO film was obtained for the degradation of organic pollutants [72]. The bandgaps of ZnO and AgCl are 3.37 eV and 3.25 eV, respectively. They could not be excited by visible light. However, after combining with Ag nanoparticles, the SPR excited electrons can transfer into the conduction band of AgCl and ZnO. Then, the photoinduced electron-hole pairs are effectively separated. Sahoo and coworkers fabricated a series of Ag@Ag_3_VO_4_/ZnCr LDH ternary heterophotocatalysts. The drastic improvement in photocatalysis is ascribed to the SPR effect and the polarization field around the Ag_3_VO_4_. The latter could accelerate the leave and transfer of photoelectrons. To maintain the energy band alignment, the energetic electrons transferred to the conduction bands of both LDH and Ag_3_VO_4_. The ternary hybrid materials may effectively separate the electron and hole pairs and thus increase the photocatalytic performance [73].

Au and Pt nanoparticles were used to modify WO_3_ nanostructures, and it has been found that Au could greatly enhance the absorption of the composite and increase the hydrogen evolution under visible light irradiation [74]. The production rate of hydrogen was measured to be 132 *μ*mol/(g·h) by the Pt/Au/WO_3_ composite, which is higher than that obtained by WO_3_. However, a substantially higher hydrogen production rate of 1513 *μ*mol/(g·h) was obtained in a TiO_2_/WO_3_/Au nanofiber system due to the plasmon enhancement of Au [75].

Weng and coworkers fabricated Pt-Au*_x_*Ag_1-*x*_-CdSe noncentrosymmetric hybrid nanostructures and utilized them in photocatalysis. They proposed a photocatalytic mechanism including three possible electron transfer pathways [76]. The plasmonic unit may enhance the photon absorption via the near-field electromagnetic enhancement [77], and before reaching the phonon-induced thermal equilibrium, the optically excited plasmons can quickly decay into hot electrons in the Fermi sea within a few picoseconds [78], which can then cross the high-energy Schottky barrier.

For the more efficient enhancement of electron-hole pairs, Mubeen and coworkers designed an effective cocatalyst system by the Au nanorods, TiO_2_, Pt nanoparticles, and Co cocatalyst for water splitting [54], which effectively utilized the hot electrons and holes induced by SPR. The electrons easily transferred to the Pt nanoparticles and the holes to the Co cocatalyst. The Au nanorod array induces the spontaneous photocatalytic water splitting reaction. Under the illumination of sunlight (100 mW/cm^2^), the system may induce approximately 5 × 10^13^ H_2_ molecules/s·cm^2^. The SPR of Au nanorods may decay into electron-hole pairs, and hot electrons may occupy the empty state of the conduction band quickly. Some of the hot electrons with sufficiently high energy may reach the surface of the Au nanorod and enter the conduction band of TiO_2_. In this system, the TiO_2_ does not participate in the photoelectronic conversion reaction but only acts as an electron filter. After passing in through TiO_2_, the electrons may be captured by Pt particles and reduced H^+^. The residual holes in the Au nanorods recombined with the electrons in the Co oxidation catalyst, and the Co oxidation catalyst could participate in the oxidation reaction of water to resupply electrons. Therefore, light is the only energy input. In this mechanism, only the electrons with sufficiently high energy may cross the Schottky barrier to be absorbed by the semiconductor and thus confine further increasing of the reaction rate [79].

## 3. Metal Cluster-Enhanced Photocatalysis

Metal clusters (usually containing tens to several hundreds of atoms) have recently attracted great attention [80]. Generally, metal nanoclusters refer to nanoparticles with a very small size that is comparable to the Fermi wavelength (the de Broglie wavelength of the conduction electrons present near the Fermi energy level). For most metal clusters (Ag and Au), the size is usually smaller than 2 nm [80]. Unlike the plasmonic nanoparticle analogue (which has a larger size), the metal nanoclusters are rather like photonic supramolecules, which exhibit discrete molecular-like energy levels and a size-dependent LUMO-HOMO gap due to quantum confinement and lose the related properties of the bulk ones, such as the SPR effect, which means the surface electrons in metal nanoclusters no longer oscillate together. Therefore, the chemical and physical properties of the metal nanoclusters are drastically changed, making the energy transfer more different and complicated from their larger counterparts. For example, the redox potential has been reported to be different. For bulk Ag metal nanoparticles in the aqueous phase, the Ag^+^/Ag redox potential is reported to be +0.79 V (vs. NHE) but is -1.80 V (vs. NHE) for Ag nanoclusters [81].

**Figure 5:**
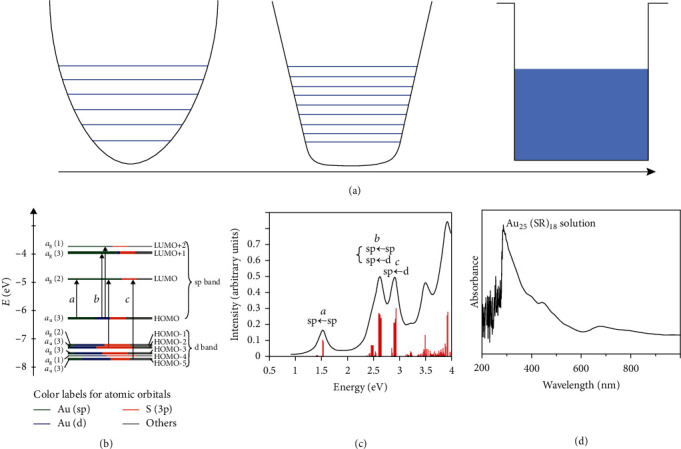
(a) Energy level diagram of Au nanoparticles with different sizes. (b, c) The energy level diagram for Au_25_(SH)_18_^−^ and the theoretical absorption spectrum of Au_25_(SH)_18_^−^ [87]. (d) The absorption spectrum of Au_25_(SR)_18_ [90].

In essence, the difference mostly originates from the variation in energy bands, which could be reflected in the optical properties. Actually, new absorption bands and emission peaks are usually found for the metal nanoclusters. In other words, such a quantum effect can greatly affect the response wavelength of light. The energy levels of Au clusters with different atom numbers are calculated and shown in Figure 5(a) [82], which could be fitted well by the energy scaling law for the clusters with atom numbers smaller than 13, indicating the spherical harmonic potential can well describe transitions of the electrons. With increasing atom numbers, the potential well of the cluster is distorted by anharmonicity into a Woods-Saxon potential surface and finally becomes a square well model for large nanoparticles. Ramakrishna and coworkers researched the absorption properties of gold nanoclusters with different atom numbers and diameters (2406 (4 nm), 976 (3 nm), 309 (2.2 nm), 140 (1.7 nm), and 25 (1.1 nm)). They found that when the number of gold atoms is higher than 976, the SPR peak can be observed. However, when the cluster number is smaller than 309, the SPR peak disappears [83]. The modulation on the size of clusters/particles opens the possibility to tweak the metal electronic states to proper levels. Berr and coworkers precisely control the atom quantity of a Pt cluster and the number of clusters combined with CdS nanorods on ITO glass [84]. By comparing the hydrogen generation with different Pt atom quantities of a cluster and the area density of the clusters on the glass, they conclude the coverage of approximately 30 Pt clusters per nanorod is sufficient. Duan and coworkers fabricated Ag nanocluster-modified TiO_2_ (P25) nanostructures [85]. Their results demonstrate a 0.89% Ag addition can greatly improve the photocatalytic efficiency of NO. The photocatalytic efficiency of the composite is approximately 3 times that of TiO_2_ and 2 times that of Ag nanoclusters. Sridharan and coworkers studied the photocatalytic activity of silver quantum clusters embedded in graphitic carbon nitride nanosheets under visible light irradiation [86]. The amount of hydrogen generated by using the composites was 1.7 times higher than that of pristine graphitic carbon nitride nanosheets.

As the most stable one among the metal clusters, Au_25_ has been demonstrated to have unique optical absorption properties (in either the visible region or the near-infrared (NIR) region) [87]. Zhu and coworkers calculated the absorption spectrum of Au_25_ nanoclusters protected by thiolate ligands [87]. Unlike the SPR nanoparticles (the absorption of light is based on the interband transition), the electronic structure in metal nanoclusters is more like molecular orbitals [88], in which the 5d^10^ and 6sp atomic orbitals constitute the HOMOs and LUMOs (Figure 5(b)). The LUMO is doubly degenerated, while the HOMO orbital is triply degenerated. After calculating with this model, the typical absorption spectrum of Au_25_(SH)_18_^−^ is shown in Figure 5(c), which agrees well with the experimental results. The peaks centered at 1.52, 2.63, and 2.91 eV were, respectively, ascribed to the LUMO ↔ HOMO transition, the mixed intraband (sp ↔ sp) and interband (sp ↔ d) transitions, and the interband transition (sp ↔ d). As a result of the quantum size effect, the electron transfer is directly influenced by the discrete energy levels, which may further influence the performance when combined with a photocatalyst [89]. The LUMO-HOMO gap of Au_25_(SH)_18_^−^ is calculated to be *E*_g_ ≈ 1.3 eV[87], indicating the Au_25_(SR)_18_ nanocluster is more like a semiconductor rather than a metal.

The photocatalytic properties of Au_25_ nanoclusters hybridized with TiO_2_ have been investigated. Yu and coworkers fabricated Au_25_(SR)_18_ nanoclusters and a nanocomposite of 99.06% TiO_2_ and 0.94% Au_25_(SR)_18_ nanoclusters (Figure 5(d)) [90]. After combining with TiO_2_ nanocrystals, the hybrid shows a 1.6 times enhancement of photocatalysis under visible light irradiation. The authors did not consider the Au_25_(SR)_18_ as an efficient electron trap to facilitate the separation of electron-hole pairs in TiO_2_. Under visible light irradiation, the Au_25_ (semiconductor) produced electron-hole pairs, and the photoelectrons can be injected into the TiO_2_ conduction band, and this has also been proved by Kogo et al. [91]. The singlet oxygen (^1^O_2_) has also been proved to be produced by Au_25_ under green light irradiation, not by TiO_2_[90].

The capping effect of the organic matter usually sophisticates the energy transfer. The protected film may not only protect the metal clusters from erosion but also affect the electron/energy transfer. One cannot exclude the protecting molecules while utilizing the noble clusters. Therefore, it is of great interest to investigate the interface properties between noble clusters and protecting molecules.

**Figure 6:**
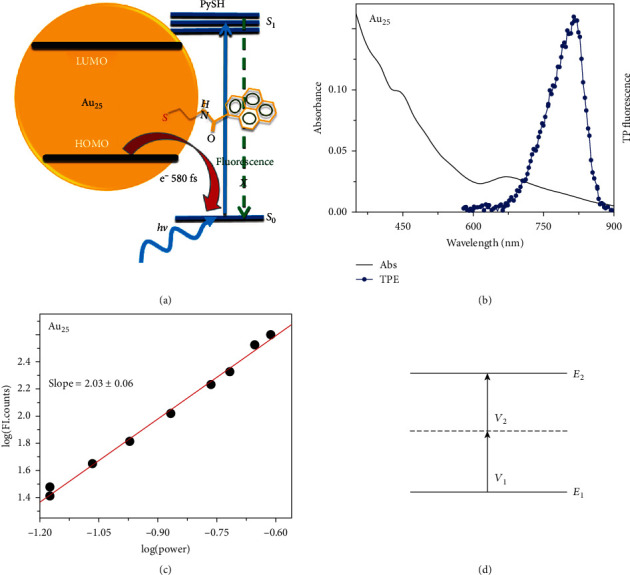
(a) Schematic of the energy transfer between Au_25_ nanoclusters and PySH [92]. (b, c) The optical absorption spectrum and two-photon emission spectrum after excitation at 1290 nm of Au_25_ clusters and the power-dependent fluorescence intensity at 1290 nm, respectively [83]. (d) The schematic of the two-photon absorption process.

Devadas and coworkers (Figure 6(a)) [92] concluded that the strong excited-state interactions between Au_25_ nanoclusters and pyrene occurred by the quenching of fluorescence, and the energy was found to transfer from Au_25_ (as electron donors) to pyrene by electrochemical investigation/fluorescence upconversion. Another work on the investigation of the photon absorption process of the Au_25_ nanoclusters is shown in Figures 6(b) and 6(c) [83]. From the pump power dependence fluorescence spectrum, the slope (FI.int) versus log(power) is approximately equal to 2, indicating a two-photon absorption process (Figure 6(d)). This means the Au_25_ could absorb two equal photons and emit one with higher energy. This property is of great value in photocatalysts, which could absorb photons with lower energy and generate electrons with higher energy. The two-photon process could be observed under excitation of 800 nm light for all the gold nanoclusters with atom numbers, even for those up to 2406.

From the abovementioned results, the modification of metal clusters with semiconductors may have more applications. Due to the restriction of the surface of semiconductors, the formed metal clusters are seldom aggregated. This makes the Au nanoclusters attractive candidates for environmental remediation as a photocatalyst or cophotocatalyst. Tatsuma and coworkers modified TiO_2_ with Au_25_ nanoclusters [88, 93]. They found that under illumination with visible and NIR light, the composite has negative photopotential shifts and anodic photocurrents, and Au_25_ showed the highest absorption efficiency among various Au clusters (Au_*n*_, *n* = 15-39). The enhancement of the TiO_2_ photocatalyst by Au_25_ nanoclusters has been explained as follows: the photons in the visible and near-infrared regions can excite the electrons of gold to the LUMO from the HOMO and leave the holes in the HOMO, which could further receive electrons from donors. The excited electrons in LUMO can inject into the conduction band of TiO_2_, which can further oxidize phenol and ferrocyanide and reduce Cu^2+^ and Ag^+^ with light of a wavelength smaller than 860 nm.

Subnanometer palladium clusters were found to be able to enhance the removal rate of NO*_x_* by TiO_2_ by 3~7 times that of bare TiO_2_ under solar light. It has been found that the Pd clusters interacted with the surface oxygen defects of TiO_2_ by an intermediate Pd oxidation state. After annealing the sample to convert the Pd nanoclusters into nanoparticles, the photocatalytic activity towards NO*_x_* decreased [94]. The utilization of metal clusters instead of SPR nanoparticles can also decrease the cost of the usage of noble metals [95].

Zhou and coworkers confined Ag clusters in the channels of ordered mesoporous anatase TiO_2_ [96]. The strong interactions of Ag atoms to oxygen atoms involved the intimate contact between Ag clusters and TiO_2_, thus increasing the electron/energy transfer between them. The hybrid shows excellent photocatalytic performance under solar light irradiation. The combination of Ag nanoclusters with g-C_3_N_4_ can achieve an 11.7 times hydrogen production over that of the neat one [97]. The increased photocatalytic activity was ascribed to the trapping effect of Ag to photoelectrons and the inhibition of the recombination tendency.

## 4. Summary and Perspectives

Metal-enhanced photocatalysis has recently been given great attention due to the accomplishment of the harvesting and transferring abilities of light energy, both in broadening the absorption wavelength range and in improving the intensity of the photocatalyst. As the metal nanoparticles contact the semiconductors, an energy barrier may form to hinder the recombination of photoinduced electrons and holes. For large metal nanoparticles (with a diameter > 2 nm), due to the SPR effect, energy can be transferred to semiconductors either by energy resonance or by hot electron injection. However, for particles that have a diameter comparable to the de Broglie wavelength, the metal nanoparticles begin to lose their metallic properties and convert into a semiconductor. Generally, the discrete energy levels endow the metal nanoclusters with fascinating properties due to the high surface energy and the small amount of utilization, but the metal nanoclusters usually need to be protected by organic matters during fabrication. As for the plasmonic particles, due to the LSPR effect, they can absorb and store the light energy and effectively transfer to the neighbors. After combining with semiconductors, both the clusters and the plasmonic particles can enhance the photocatalytic properties, either in enlarging the light absorption region or in enhancing the activity of electrons/holes. The properties of metal nanoparticles severely relied on the band structure, surface atoms, crystal structure, exposed facets, etc. Although great progress has been achieved in this area, further systematic investigations are required to deeply address the mechanism of energy transfer between metal nanoparticles and semiconductors or target molecules. The existing results are still unsatisfactory, especially on the energy transfer process; many of them lack direct observation and are full of confusion. The investigation into the metal-enhanced photocatalysis is now mainly focused on the SPR effect. The effect of the metal clusters on the enhancement of photocatalysis is relatively scarce, making the investigation and application of metal clusters not sufficient as the SPR effect.

Regardless, there are some topics we think are challenging and of interest for future development:

*(1) The Charge/Energy Transfer at the Metal and Semiconductor Interface*. This is of interest for explaining the enhancement mechanism. Up to now, the mechanism is still not very clear, and lots of explanations on the enhancement are still based on indirect proofs. Therefore, new ideas and new techniques on the direct characterization of the separation and transfer of electron-hole pairs are still full of challenges. At the same time, the multicomponent combinations have indicated an effective decrease in the recombination efficiency of electron-hole pairs, but too many components may increase the carrier scattering at the interface, which is a negative effect for photocatalysis. Adopting a rational component type, structure, fabrication method, and number is full of challenges and interests.

*(2) The Effect of Metals on the Catalytic Reaction*. Actually, the metals combined with semiconductors may influence the path of photocatalysis. Although it is not researched very well, it is another unquestionable major issue. The photocatalytic reaction may be greatly influenced by the metal particles (the size, exposed surface, component, crystallization, etc.). Construction of the relationship between the metals combined with semiconductors and the photocatalytic reaction is one of the grand challenges and must stimulate long-lasting scientific and technological interests in the field.

*(3) The Energy Transfer between Metals and Semiconductors*. Although the metal nanocluster-enhanced photocatalysis has started to be of concern, some works demonstrate the energy transfer between metals and semiconductors because the energy levels of the clusters may be severely changed by adding or removing one atom. This makes it difficult because precisely controlling the number of atoms needs high experimental techniques. Therefore, the size control of the metal particles, especially the nanoclusters, becomes one of the important and challenging research fields.

*(4) The Effect of Metals on the Photocatalytic Reaction*. The catalytic reaction, especially the reaction path, is an important aspect of designing the highly efficient photocatalyst. However, the research on the effect of metal clusters/nanoparticles on the reaction path is seldom related.
